# Evolutionary Implications of Mechanistic Models of TE-Mediated Hybrid Incompatibility

**DOI:** 10.1155/2012/698198

**Published:** 2012-02-16

**Authors:** Dean M. Castillo, Leonie C. Moyle

**Affiliations:** Department of Biology, Indiana University, 1003 Third Street, Bloomington, IN 47405, USA

## Abstract

New models of TE repression in plants (specifically *Arabidopsis*) have suggested specific mechanisms by which TE misregulation in hybrids might result in the expression of hybrid inviability. If true, these models suggest as yet undescribed consequences for (1) mechanistic connections between hybrid problems expressed at different postzygotic stages (e.g., inviability versus sterility), (2) the predicted strength, stage, and direction of isolation between diverging lineages that differ in TE activity, and (3) the association between species attributes that influence TE dynamics (e.g., mode of reproduction, geographical structure) and the rate at which they could accumulate incompatibilities. In this paper, we explore these implications and outline future empirical directions for generating data necessary to evaluate them.

## 1. Introduction

In many plants and animals, hybrid inviability (lethality) or hybrid sterility act as postfertilization barriers to hybridization [1]. These incompatibilities can be explained by negative genetic interactions between two or more loci under the Dobzhansky-Muller model of hybrid incompatibility [2, 3]. The Dobzhansky-Muller model does not specify the nature of the genetic elements that can lead to hybrid incompatibilities, and to date there are few cases where the loci responsible have been directly identified [4, 5]. The idea that transposable elements (TEs) can influence hybrid sterility is well established in *Drosophila* and has been highlighted in studies of intraspecific crosses resulting in hybrid dysgenesis [6–8]. However, since TE mobilization in hybrids between animal species is less frequently observed than in plants [9–11], skepticism has arisen surrounding the role that TE movement plays in the evolution of reproductive isolation [1, 11]. Recent epigenetic studies in plants, however, are providing evidence that a common genetic pathway, involving siRNA regulation of transposable elements, might lead to both hybrid inviability and hybrid sterility [12–14]. In this paper, our goal is to examine implications of these mechanistic models for the genetics and evolution of reproductive isolation due to TE misregulation.

**Figure 1:**
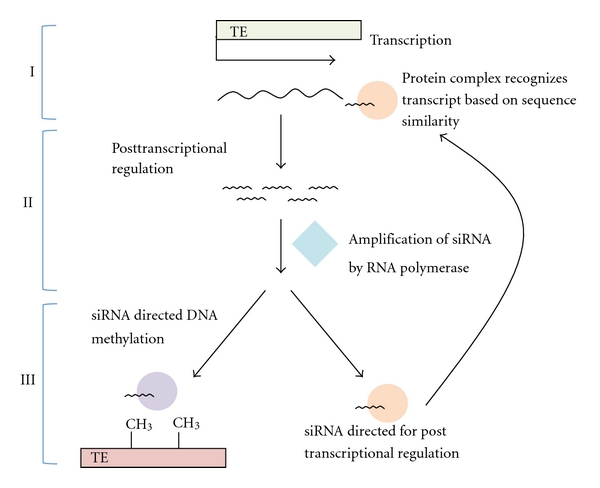
A general model of the siRNA pathway demonstrating the three processes observed in both plants and animals. (1) Transposon transcripts are detected by complementary small RNAs derived from previously transcribed transposons. (2) These transcripts are post-transcriptionally cleaved by small RNA-protein complexes creating small RNAs that are amplified through an RNA-dependent RNA polymerase. (3) These newly derived small RNAs are used to target transposon transcripts as in step (1) or target the transposon sequences in the genome to induce DNA methylation and repressive chromatin modifications.

Transposable element suppression in somatic cells is generally conserved among all organisms and requires three steps [15], see Figure 1. (1) Transposon transcripts are detected by complementary small RNAs derived from previously transcribed transposons (typically siRNAs in plants and piRNAs in animals). (2) These transcripts are posttranscriptionally cleaved by small RNA-protein complexes creating small RNAs that are amplified through an RNA-dependent RNA polymerase. (3) These newly derived small RNAs are used to target transposon transcripts as in step (1) or to target the transposon sequences in the genome to induce DNA methylation and repressive chromatin modifications. Slight modifications to the pathway occur during regulation of TEs in germ line cells.

Small RNA pathways have been proposed to play a role in regulating imprinted genes and genome-wide methylation patterns; this role may influence gene expression in hybrid individuals. In this review, however, we will focus on TE regulation during the development of male and female plant gametophytes, and the consequences this could have for the expression of postzygotic hybrid incompatibilities. First, we briefly describe the current mechanistic models of TE regulation in gametogenesis, including prior connections that have been drawn between TE misregulation and the expression of hybrid incompatibilities, and indirect evidence for this association in plants (i.e., *Arabidopsis*). Second, we discuss unexplored consequences of these emerging models for the genetics and evolution of species barriers based on this mechanism. Based on this, we identify several implications of evolutionary importance: (1) TE-misregulation is a mechanism that can produce nonindependent accumulation of hybrid problems at different developmental stages of isolation between species (e.g., hybrid inviability and hybrid sterility). (2) Where this mechanism is an important contributor to reproductive isolation, the direction and strength of hybrid problems should be predictable based on lineage differences in TE abundance (“load”) and sequence identity. (3) Factors influencing the divergence of TEs among lineages should also influence when and where TE-mediated hybrid incompatibility is most likely to be observed. We identify empirical data that will be essential to assess these inferences in the future, and potential approaches for generating these data. Finally, we sketch some similarities and differences between plants and animals that could affect hybrid problems related to TEs in these groups. One of our goals is to anticipate patterns of reproductive isolation that might operate at different stages or under different ecological or evolutionary scenarios, if TE misregulation (as described by these mechanistic models) is an important contributor to reproductive isolation.

## 2. Mechanistic Models of TE Suppression during Male and Female Gametogenesis

Recent findings have prompted Slotkin et al. [16] to propose a companion cell model for TE regulation in male (pollen) gametogenesis that is similar to a model of piRNA accumulation in *Drosophila* egg development [17]. In angiosperm male gametes, the growing (postpollination) pollen tube contains three nuclei; two are sperm nuclei that will be involved in fertilization (see below), while one is a vegetative nucleus that does not fuse with the maternal gametophyte [18]. In the Slotkin et al. [16] model, the vegetative nucleus of pollen acts as a companion cell that “sacrifices” itself so that the sperm cells can maintain correct TE suppression. In this nucleus, TEs are demethylated, increasing transcription of elements (most that are normally somatically silenced). These transcripts are posttranscriptionally processed, creating a pool of small RNAs. These small RNAs are transported to the sperm nuclei where they direct methylation of TEs, effectively “resetting” the appropriate suppression of TEs in the germline nuclei prior to fertilization. The transcription of TEs in the pollen vegetative nucleus is initiated after the loss of heterochromatin and MET1 (cytosine-DNA-methyltransferase) and DDM1 (decreased DNA methylation) proteins [16], indicating an actively regulated process that is specific to this nucleus only. The small RNAs produced in pollen are predominantly 21-nt long and, besides translocation to the sperm cell, these small RNAs are also delivered into the zygote and endosperm [16], leading to potential consequences for regulation, and misregulation, at postfertilization stages.

Similar to pollen development, the TE-derived small RNAs necessary for TE regulation in the female germ line (gametophyte) are created in an adjoining cell that will not contribute DNA to the next generation (in this case, epidermal cells lining the ovary) [19]. In angiosperms, the mature female gametophyte is composed of seven cells containing eight genetically identical haploid nuclei (all the products of a single initial meiotic product), surrounded by maternal (sporophytic) tissue. Two of these cells will contribute tissues to the F1 following typical “double fertilization”: the haploid ovule (egg) fuses with one sperm cell to give rise to the developing embryo; the doubled-haploid (2 × 1N) “central cell” fuses with the second sperm cell to give rise to the 3N endosperm—a nutritive tissue that accumulates resources postfertilization to support development of the embryo. During development of the female gametophtye, small RNAs are translocated from the “sacrificial” epidermal cells into the ovule and central cell; these act to silence TEs in the developing embryo and endosperm after fertilization [20]. Another specific component of TE regulation, the Argonaut protein AGO9, is also expressed in the maternal sporophytic cells, but not in germ cells, and interacts preferentially with the TE-derived 24-nt small RNAs to suppress TE activity in the female gametophyte [19].

## 3. Connections between TE Misregulation and Hybrid Incompatibility

Based on these emerging mechanistic details, several models have recently been proposed connecting TE regulation and hybrid failure. These models have focused primarily on seed failure via misregulation of development in the endosperm [12, 13, 21], partly because some of the most evident hybrid incompatibility phenotypes in *Arabidopsis *involve endosperm failure in interspecies and interploidy crosses. Both types of crosses give rise to seed collapse (inviability) due to either overproliferation and failure to cellularize, or under-proliferation and premature cellularization, in the hybrid endosperm [22–24]. Because these phenotypes are reminiscent of the examples of overgrowth in *Peromyscus* [25, 26], earlier models hypothesized that hybrid failure results from misregulation of imprinted genes. Recently, however, Martienssen [13] proposed an alternative model where mismatches in small RNAs contributed by each parent are responsible for endosperm failure, based on the evidence that TE sequences and TE abundance differ substantially between the *A. arenosa* and *A. thaliana* genomes [21, 27]. Martienssen [13] suggests that if TE sequences in the central cell do not match siRNA in the pollen or, reciprocally, if siRNA from the female germ line do not match TE from the sperm, then TEs will be active in the endosperm causing endosperm failure. Martienssen's model does not specify whether small RNAs have to match in terms of sequence similarity or overall quantity, as the crosses on which his model is based (*A. thaliana *by* A. arenosa*) potentially involve both differences in quantity and sequence.

The idea of an interaction between male and female “factors” controlling endosperm development has been used previously in the *Arabidopsis* literature. For example, Josefsson et al. [21] proposed a model—“dosage-dependent induction”—to explain endosperm failure in terms of the interactions between maternal and paternal factors that they observed in *Arabidopsis* hybrids, though they did not implicate TE-derived small RNAs. In their model, it is the sole responsibility of the maternal parent to deliver the proper number of “repressors” to saturate target sites in the maternal genome as well as target sites contributed by the paternal genome. If the total amount of repressor is insufficient, both maternal and paternal target sites will escape silencing. Conversely, extra doses of maternal genome can “rescue” an otherwise incompatible cross by increasing the total number of repressors. This model is generally consistent with longstanding observations in numerous species crosses, where simply manipulating maternal dosage can alter the compatibility of a cross (e.g., [28, 29]). The model is fundamentally different from the Martienssen [13] model because the male only contributes targets that require silencing but is not able to silence TEs that it contributes or that the maternal parent contributes. Michalak [12] has reinterpreted the “dosage-dependent induction” model in terms of small RNAs. In his example, he assumes that if there is a difference in the deposition of maternally loaded small RNAs between species, or if these small RNAs differ in their capabilities to suppress their targets, TEs can become active in hybrid crosses. The activation of TEs can cause endosperm failure, thereby acting as a reproductive barrier.

In both models, hybrid incompatibility is a property of TEs being preferentially activated in the endosperm, with no focus on TE activation in the embryo. One compelling reason that TE activation might preferentially occur in the endosperm is the empirical observation that the endosperm is loaded with RNAs by both parents [13], and gene regulation in the endosperm is, therefore, potentially influenced by both parental genomes. The embryo, in contrast, does not play a large role in early seed development and is less influenced by parental provisioning as most gene expression beyond the first few cell divisions is regulated within the embryo itself [30].

Both models provide new testable predictions and have the potential to provide a mechanism for earlier models of endosperm failure (e.g., the “endosperm balance number” model; [28]), but these models currently have a limited scope as general explanations of hybrid failure between species. In particular, they do not address expectations or predictions with respect to (a) the expression of hybrid failure at several other stages of reproductive isolation; (b) the differential effects of lineage differences in TE/small RNA abundance (“load”) versus TE/small RNA sequence identity; (c) the evolutionary/ecological factors that can influence TE dynamics, and therefore the expected accumulation of isolation between linages that are differentially affected by these factors. Here, we address these different factors and some of their implications for the evolution of isolating barriers.

### 3.1. Broader Applications: TE-Mediated Hybrid Incompatibility at Other Isolation Stages

Although current plant models have focused on seed failure (endosperm breakdown in early F1 seed), there is reason to believe that inappropriate TE mobilization could also influence other isolation stages, including F1 male and female sterility. Indeed, the two models we have discussed are very similar to the piRNA models of transposon silencing in animals because they assume that TEs are reactivated in specific accessory cells to enhance small RNA-triggered silencing of TEs in germ line cells [20]. The phenotype observed in *Drosophila* studies of TE-mediated “hybrid dysgenesis” is male sterility. Hybrid sterility (in the form of pollen sterility) has also been observed in crosses between diploid *Arabidopsis* species [31], and Martienssen [13] discusses how the dysgenesis model could also be applied to pollen sterility in *Arabidopsis*. In addition to F1 hybrid male sterility, hybrid female sterility is also a potential consequence of TE-misregulation in a hybrid genome. For example, in the female gametophyte, proper regulation of TEs has been associated with restriction of cell fate [19]; if TEs are not correctly suppressed in the megaspore mother cell, aberrant phenotypes (including two female gametophytes in a single ovule) can occur, leading to female sterility. Finally, mutant studies indicate that misregulation of small RNAs has the potential to prevent proper double fertilization [32], although this empirical example does not involve TE-derived small RNAs.

 These examples suggest that TE-mediated hybrid incompatibilities could occur at a range of stages, from early F1 inviability to F1 sterility. Given this, are there consequences for our understanding of the evolution of hybrid incompatibilities? TE-based models suggest a direct mechanistic connection, and therefore nonindependence, between different stages of postzygotic isolation in a single cross. This is inconsistent with most current models of the evolution of hybrid incompatibility, which assume independence among fixations contributing to different stages of isolation [33]. This independence assumption is reasonable for many loci contributing to species barriers, whose hybrid incompatibility effects are thought to be incidental by-products of evolutionary divergence at loci from many different potential developmental or reproductive processes [1]. However, the tight mechanistic connection between regulation of TEs during different developmental stages indicates that this independence assumption is likely violated, and therefore that predictions about hybrid incompatibilities that rely on this assumption might not hold for TE-mediated hybrid incompatibility. For example, under the Dobzhansky-Muller model of hybrid incompatibilities, the number of reproductive isolation loci is predicted [34] and observed [35, 36] to “snowball” (i.e., increase faster than linearly with time) between diverging lineages. The snowball prediction emerges from the condition that each new fixation within a diverging lineage can potentially interact with every other evolutionary change that has preceeded it during lineage divergence [33, 34]). When fixations producing reproductive isolation instead involve interactions between a limited, nonindependent set of loci, these predictions do not hold (e.g., [37]). We suggest, then, that TE-mediated incompatibilities might not follow the predictions of such models. Instead, TE-mediated incompatibilities might behave similarly to other “conflict” driven hybrid incompatibilities [4], where reproductive isolation is due to interactions between a small subset of loci evolving according to antagonistic coevolutionary processes (see further below).

### 3.2. Differentiating the Contribution of Divergence in TE/Small RNA Quantity versus Sequence Identity

**Figure 2:**
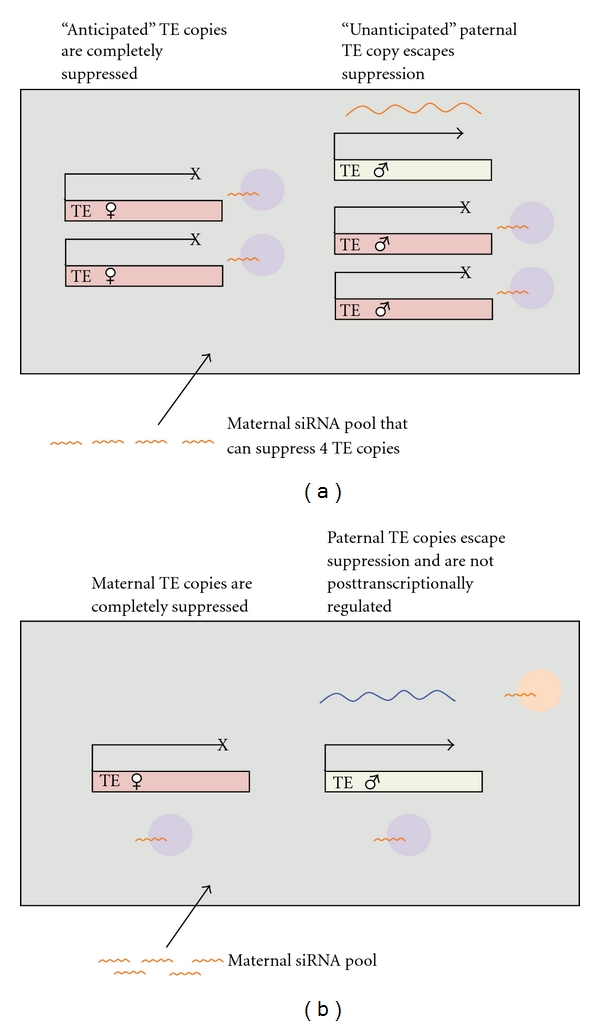
The misregulation of TEs due to a mismatch of maternal siRNA and paternal TE copies, consistent with the Martienssen and Josefsson models of endosperm failure. (a) TE load differences between parents: the maternal siRNA cannot suppress paternal TE copies due to excess of paternal TE copies. (b) TE sequence mismatch between parents: the maternal siRNA cannot suppress paternal TE copies due to differences in sequences of the TEs.

Current models of hybrid failure are coy about the range of mechanisms by which maternal/paternal “mismatch” in TE regulation can occur. In the *Arabidopsis* interspecific crosses, it is clear that the abundance of TEs—either TE copy number or size of the resulting small RNA pool contributed by the paternal and maternal genomes—could determine the outcome of the cross, as both are correlated with endosperm failure. In this case, stoichiometric mismatch is responsible for the inappropriate regulation of either gametogenesis or postfertilization development (Figure 2(a)). Alternatively, parental “mismatch” could be due to sequence divergence between TEs in different lineages, whereby small RNAs generated from one lineage may fail to recognize or to effectively interact with target sequences from the alternative lineage, due to base-pairing mismatches (Figure 2(b)). Although there is no direct empirical evidence, it has been suggested that sequence specificity plays an important role in TE suppression [38, 39] and the proteins that interact with small RNAs rely on sequence complementarity to target TEs for methylation and silencing [40]. Given this, transcripts from one TE copy may not be able to target slightly different TE copies, although currently it is not clear how much sequence divergence must occur before TE copies can no longer suppress one another.

Regardless, determining the relative effect of TE abundance (“load”) versus sequence divergence on hybrid incompatibilities might be important because, as we argue here, these two different forms of divergence are expected to be more influential at different stages of reproductive isolation. These expectations can be inferred from the current mechanistic models of TE suppression at different developmental stages in plants. For example, TE silencing during prefertilization gametogenesis and postfertilization endosperm development both rely on sequence complementarity between small RNAs and target sites [38, 39]. However, these two developmental stages differ in that silencing in the endosperm is dependent on parentally derived small RNAs whereas silencing during gametogenesis is regulated by self-generated small RNAs. This difference in the source of siRNA regulators creates the potential for different kinds of TE divergence to differentially affect early F1 viability following heterospecific fertilization, versus gametogenesis in an F1 hybrid individual and/or hybrid problems in later generation (e.g., F2) individuals. 

First, consider the F1 offspring from an interspecific (hybrid) cross. In the developing hybrid endosperm, small RNA sequences from the maternal/paternal genome will not necessarily match target sites in the other (heterospecific) genome, but they will be capable of silencing TEs derived from their own (homospecific) genome. Some of this TE suppression takes place prior to fertilization, that is, in the sperm cells within pollen and in the female gametophyte (see above). Other regulatory control relies on postfertilization suppression, where it appears that the primary (but likely not sole; [16]) determinant of control is maternally loaded siRNAs. This “asymmetry” in control is important for the expression of hybrid problems in the early developing offspring. In particular, if the maternal parent contributes a quantity of small RNAs sufficient to regulate TEs in its own (diploid) genome, but not in excess to accommodate extra “unanticipated”copies from the other genome, TE suppression in the postfertilization offspring will be incomplete. This specifically occurs when a “low load” lineage acts as the maternal parent in a cross with males from a “high load” lineage. Large differences in TE copies among parental genotypes could also be magnified during the production of small RNAs, if this is nonlinearly related to TE copy number.

Note that differences in sequence identity might exacerbate these load differences; when either parent is lacking copies of a specific TE, the endosperm might be overwhelmed with TE activity. Again, the expectation is that this will be observed when “naïve” lineages act as the maternal parent in crosses to males from lineages that contain the novel TE, but not necessarily in the reciprocal cross. Regardless, it appears that differences specifically in TE load are most likely to be influence misregulation of TE suppression in early F1 (e.g., endosperm) development. Note that details of this prediction rely on the supremacy of maternally loaded siRNAs during early offspring development; if paternally loaded siRNAs are as important as maternally loaded siRNAs, some expectations might not hold. For example, in the Martienssen [13] model, either parental genome can be “overwhelmed” whereas in the Josefsson et al. [21] model only the maternal genome has the potential to be overwhelmed.

Second, consider the fertility of F1 offspring from an interspecific (hybrid) cross. For TEs to cause hybrid sterility, they must be improperly regulated during gametogenesis in the hybrid individual. This might occur if the small RNAs created during gametogenesis cannot fully silence TEs inherited from both parents. F1 hybrids will have all possible target sites and small RNA-producing loci, so how could incomplete suppression occur in F1s? One possible circumstance is if siRNA-mediated TE suppression is haploinsufficient (either due to siRNA production or TE targeting), for example, if there is a nonlinear relationship between siRNA suppression efficacy and TE copy number. Note that this is more likely to be due to differences in TE sequence identity among parental lineages; happloinsufficiency applies only to those loci for which the F1 is functionally haploid (i.e., loci missing in one parental lineage). In comparison, differences in load may not be important during hybrid gametogenesis because F1 individuals would have a haploid complement of TE copies from both parents; thus there are no “unanticipated” TE copies to be accounted for, unlike during endosperm TE suppression. Regardless, the expression of F1 sterility due to divergence in TE identity is expected to be “symmetric;” reciprocal F1s should show the same sterility effects.

 Finally, consider the viability and fertility of recombinant later generation (e.g., F2) hybrids. In addition to the above effects, in these individuals the potentially independent segregation of TEs and their regulators is important. In animals, small RNAs used for TE suppression are primarily derived from TE clusters located in (sometimes distant) heterochromatic regions [41]. TEs in these clusters are generally no longer active and as a result their DNA sequence can change rapidly. If the hybrid progeny does not inherit these clusters (even if they have inherited other TE copies), or if sequences in the clusters no longer match the active copies outside of the cluster, then they might not generate sufficient small RNAs. If the small RNA cluster model is not appropriate for plants, variation in the efficiency of producing small RNAs may still exist between TE copies and could influence the production of small RNA pools.

 Overall, on the basis of these mechanistic models, we infer that TE “load” (copy number) differences are likely more important at early (embryonic) stages of hybrid formation, when parentally (especially maternally) loaded siRNAs are critical for TE regulation. In comparison, differences in TE identity are likely more important for the expression of hybrid sterility. In addition, the symmetry of isolation between reciprocal crosses is expected to differ between these stages. Given this, depending on the factors that differentially affect these modes of TE divergence, different expression of TE-mediated hybrid incompatibility might be expected under different ecological and evolutionary scenarios.

### 3.3. Evolutionary Expectations from Predicted TE Dynamics

Evolutionary models have already been used to describe and predict TE dynamics within and between populations. Understanding the forces affecting TE outbreaks, proliferation, spread, and suppression might therefore help in predicting conditions under which lineages can differ in TEs, and therefore when TEs are most likely be involved in species barriers between them.

#### 3.3.1. Factors Influencing Evolutionary Divergence in TE Abundance and Identity

For TE misregulation to influence the expression of hybrid incompatibilities, diverging lineages must differ in the activity and/or identity of their TEs. What conditions influence the accumulation of different TE copy number (load) or novel TE sequences among lineages? TE dynamics have been examined extensively using population genetic models, especially to understand conditions that produce a stable transposition-selection balance [42]. Some models have incorporated host responses in the form of alleles that suppress activity of new TE invaders [43], although we are aware of only one model that specifically incorporates the possibility of sRNA-mediated TE suppression [44]. Drawing from these models, Blumenstiel [45] has summarized the four phases of TE invasion in host populations (and the mechanisms/factors that influence these four phases): (i) invasion of a new TE (either via mutation of an existing TE, or via horizontal transmission); (ii) TE proliferation, polymorphism, and fixation in the host population (where the rate and extent of proliferation depends on transposition rates, selection against the negative effects of TEs in hosts, migration rates in the host species, and drift); (iii) the origin of a repressor locus (via a new insertion (mutation) with repressive effects); (iv) fixation of the repressor allele, and decay of the new TE family. The completion of phase IV can depend on the degree of linkage between the repressor and its targets, and whether there are other collateral effects of silencing on host function; under some conditions, the repressor allele is not expected to fix (see [45], and references therein).

Perhaps unsurprisingly, then, the factors affecting population differences in the proliferation and/or divergence of TEs are the standard evolutionary forces of mutation, migration, drift, and selection. Clearly, however, the relative influence of these factors could be affected by whether hosts can respond to new TE outbreaks by fine-tuning existing sRNA mechanisms of control [45]. For example, the (mutational) origin of a new suppressor allele becomes less important if hosts have a preexisting mechanism for suppressing the negative effects of novel TEs they encounter. Preexisting small RNA pathways might also influence the strength of selection against potential deleterious effects of TE proliferation. Indeed, models indicate that TE insertions that are the target of RNA-mediated silencing are more likely to drift to higher frequency (because their deleterious effects are moderated via this host control) than insertions that are not targets of RNA-mediated silencing [44].

Notwithstanding such observations, some factors seem more predictably associated with TE proliferation and spread regardless of, for example, the mechanism of TE control. In particular, migration can directly influence the rate at which TEs spread between populations. When there is gene flow, populations with different TE copy number can become homogenized because TEs have the potential to invade “empty” populations. In highly structured populations, however, the homogenization process is slow; as a result, heterogeneous TE copy number distributions (and, presumably, TE identities) can be maintained over long periods of time when TE proliferation is different between populations [46].

Migration or population subdivision will be influenced by life history and demographic characteristics of the host species. For example, on average, species with active dispersal mechanisms are expected (and observed; e.g., [47]) to be less subdivided than passively dispersing species. The magnitude of population subdivision can also be strongly influenced by the mode of reproduction. In plants, for example, selfing species are more strongly genetically subdivided than mixed maters or outcrossers (e.g., [47]). The mode of reproduction also influences effective population size (i.e., N_e_ is smaller in selfing species) and therefore the relative influence of drift on TE dynamics. Drift can act to magnify population differences in TEs by allowing the stochastic accumulation of TE load [48, 49].

These observations suggest that TE accumulation might be expected to be greater among selfing lineages. However, some models indicate that the influence of mating system on TE copy number is dependent on the specific mode of selection acting against TEs [50, 51]: where selection acts on the deleterious effects of TE insertions, both the probability that a TE is lost when its initial frequency is low and the TE copy number increase as self-fertilization increases; when selection acts on the effects of ectopic recombination between TE copies, the exact opposite effects are observed. Interestingly, several studies that have compared TE insertion frequency in selfing and outcrossing lineages produce inconsistent results as to whether selfing lineages have lower or higher TE copy number [52–55]. Also, there is no clear evidence that TE copy number and self-fertilization rate are correlated [51]. This might suggest that the most pertinent factors for TE accumulation and differentiation are N_e_ and migration, parameters that are only imperfectly correlated with mode of reproduction.

#### 3.3.2. Evolutionary Expectations about the Accumulation of TE Differences

Given these complexities, based on the current models it is difficult to draw many strong generalizations about the factors that can influence the origin and spread of TEs, especially because it remains unclear how selection acts on TEs [56, 57], including those that are subject to siRNA-mediated repression [44] (and see below). Further work will be necessary to clarify this in the future. Nonetheless, it seems likely that factors that influence the frequency with which new TE outbreaks occur (such as low N_e_ that reduces the efficiency with which TEs are suppressed) and the degree to which individual TE outbreaks are locally “quarantined” (such as the extent of population subdivision/migration) can influence the rates at which TE differences are able to build up between lineages. Similarly, factors that influence the rates of sequence evolution of TEs should also influence TE divergence. For example, antagonistic selection, including intergenomic conflict, has the potential to cause rapid divergence between populations [58]; thus conditions that contribute to the efficacy of antagonistic selection (such as the ability of parasites to escape from their own deleterious effects, via horizontal transmission) could also elevate the rates of lineage differentiation in TEs.

By influencing the accumulation of TE differences, such factors could influence the relative contribution of TE misregulation to the expression of species barriers among lineages. This is because these factors are not necessarily equally favorable for the fixation of other (non-TE) genetic differences that can also contribute to reproductive isolation. For example, as genomic parasites, TEs are able to “invade” empty populations (even when they have some fitness costs for their hosts), making quarantine fundamentally important in controlling TE homogenization. In comparison, complete population subdivision is not essential for adaptive genetic differentiation between populations, which can be more dependent upon the strength of local selection against immigrants. Similarly, conditions favoring antagonistic coevolution need not be the same as those favoring other adaptive fixations. Synergism among such factors might be especially favorable to TE differentiation. For example, under a geographic mosaic model, population subdivision and local (antagonistic) selection could jointly contribute to divergence in TE load and sequence identity [59, 60], although this is also true of any other potential isolating factors that are also subject to antagonistic coevolution. Regardless, given the historical attention to TE dynamics, it is surprisingly difficult to make strong predictions about factors expected to promote the accumulation of TEs between species. In future, perhaps the most clarity will not come from theoretical approaches, but rather from more taxonomically diverse and replicated empirical comparisons of TE differentiation between lineages that differ in key biological features.

#### 3.3.3. Possible Targets of Evolutionary Change and Causes of RI

Even with limited clarity about evolutionary conditions favoring TE differences, there are some suggestive evolutionary targets that might be responsible for lineage differentiation in TE proliferation and control. For example, under the companion cell model, TEs must move from somatic cells to the germ cells to ensure they are passed to the next generation. It is at this stage of development that the interaction between TEs, suppressor alleles, and the small RNA pathway can be subject to strong selection [44] and therefore potentially rapid lineage divergence. Active TEs can only escape suppression if their sequence is divergent from small RNA producing loci or, if they produce small RNAs, when these RNAs do not interact efficiently with proteins in small RNA pathways. If small RNA pathways are under strong selection to suppress TEs in their genome, antagonistic selection from TEs trying to escape suppression can cause arms race dynamics. The piRNA machinery in some *Drosophila* has been shown to be under strong positive selection, implying a potential arms race (but see [61]), although it has yet to be shown that selection is acting on TEs to escape suppression [62]. It is known in animals that Argonaut and Piwi proteins rely on sequence complementarity to direct TE methylation and suppression. If these proteins are fine-tuned to target specific TEs, then they may be less efficient at interacting with small RNAs from novel TEs and therefore at silencing these TEs.

Other potential targets of selective differentiation are host regulators of small RNA pools. For example, a general step in the small RNA directed TE-silencing pathway is amplification of small RNAs by an RNA-dependent polymerase. In *Arabidopsis*, the main polymerase involved in RNAi activities, including TE suppression, is Pol-IV [63]. As Pol-IV is not necessary for survival, selection acting on Pol-IV might be similar to forces affecting piRNA machinery in animals, including specialization for the TEs that occur in a specific genome; in this case, Pol-IV in hybrids may not be able to efficiently process novel small RNAs. Interestingly, changes in these targets might also result in more “standard” Dobzhansky-Muller type incompatibilities. RNA polymerases typically consist of 12 subunits, and specialization in different lineages might render subunits incompatible with their counterparts from different lineages when brought together in hybrids. In this scenario, incorrect enzyme assembly might disrupt protein function, similar to the description of PcG complex dynamics by Ishikawa and Kinoshita [64].

## 4. Future Empirical Approaches Linking TE Misregulation to Hybrid Incompatibility

To date, models inferring the involvement of TE misregulation in hybrid incompatibility are more suggestive than definitive, and unambiguous evidence connecting these phenomena has yet to be shown. Indeed, the specific molecular mechanisms by which TE derepression might cause cell death (and therefore hybrid problems) remains unknown, and there is currently no published evidence that mechanistically links inactivation of pathways that regulate TE suppression with hybrid sterility or lethality. In some cases, there is evidence that TE derepression has no significant fitness effect in hybrids. Chen et al. [27] have demonstrated that RNAi knockouts for met1 in *Arabidopsis suecica* do exhibit increased levels of TE expression but sterility is not observed in these individuals and the increase of TE expression is not seen in resynthesized allotetraploids. Similarly, maize lineages appear to have large differences in TE content and abundance [65] but crosses among them do not show evidence of deleterious incompatibility phenotypes (e.g., [66]). Indeed, it is unclear whether mobilization *per se* should be predominantly responsible for hybrid incompatibility phenotypes; for example, an alternative mechanism is that TEs indirectly affect incompatibility through the collateral misregulation of genes that have acquired TE regulatory elements or of genes adjacent to these loci [67]. Ultimately, these are questions that must be addressed if the aim is to truly assess the evolutionary importance of TE-mediated isolation mechanisms.

Given this lack of empirical data, to demonstrate that TE misregulation is responsible for hybrid incompatibilities in any given system, several pieces of empirical evidence will be essential. First, lineages must differ in TE and small RNA load and/or identity, and in the parental contributions of these to offspring; if they do not, divergence in TEs clearly cannot be responsible for hybrid problems. Second, lineage divergence in TE load or identity must be consistently associated with the phenotypic expression of postzygotic isolation, such as, reproductive and developmental problems in hybrids. Finally, the expression of specific reproductive isolating barriers must be functionally linked to TE derepression. Fortunately, in combination with classical genetics, several emerging empirical approaches now make these pieces of evidence attainable now or in the near future. These data will also be useful in evaluating some of the expectations we have identified above.

### 4.1. Characterizing Lineage Differences in TEs and Parental Contributions of Small RNAs

Traditionally, quantifying genotype differences in TEs required approaches such as “transposon display,” where selective PCR produces a fingerprint of TE insertions for each genotype [68]. This approach can be used to roughly quantify relative copy number and describe differences between host lineages, for a known TE family [52, 53, 69, 70]. Increasingly, however, whole genome sequencing is being used to describe the entire suite of TEs within a given genome [71, 72]. This approach is not limited to known TE families and therefore can, in principle, detect TEs with novel sequences in addition to copy number differences between different genomes.

 In addition to describing TE load and sequence differences between lineages, next generation deep sequencing of small RNA pools is also now routinely used to quantify tissue-specific small RNAs and changes in small RNA production in mutant lines [73–76]. An understanding of each parental contribution of both TE copy number (as a measure of load) and small RNAs would be necessary to examine the predictions of the Josefsson et. al. [21] and Martienssen [13] models. For example, the main prediction of the Martienssen model is that endosperm failure will occur when maternal and paternal small RNAs do not match the TE sequences contributed by the other parent. Similarly, in the Josefsson et al. [21] model, differences in maternal small RNA contributions will determine endosperm development. Deep sequencing of small RNA pools in pollen, female gametophyte, and endosperm will allow researchers to identify which small RNAs are contributed by each parent. Small RNAs could be mapped to genomic or EST sequences to determine the identity of small RNA producing loci in the maternal and paternal genomes [41, 77]. As assembly algorithms improve for short sequences, small RNAs could be assembled *de novo*. Copy number could be assayed using inverse PCR techniques or qPCR based on sequences uncovered by deep sequencing.

Next generation sequencing, therefore, provides the technology necessary for describing lineage and parental differences in TEs and small RNAs, and for comparing hybrid small RNA pools to equivalent parental pools. Nonetheless, some limitations remain. As genome sequencing technology improves, sequencing small RNAs and whole genomes will become easier but alignment and assembly is still a limiting step for repetitive elements. In addition, quantification of small RNAs can give an estimate of the total size of the small RNA pool, but determining the quantity contributed by a single TE is not yet achievable. Moreover, if the TE family is young and different insertions have identical DNA sequences, quantifying and identifying small RNAs to a specific TE locus is not possible. In plants, it is thought that all TE copies contribute to small RNAs, but the model emerging in animals is that clusters of mostly nonactive TEs contribute to the small RNA pool to target active TEs [41]. These challenges will need to be tackled by technological and bioinformatic advances in the future.

### 4.2. Demonstrating the Association between Isolating Barriers and TE Differentiation

To date, an association between differences in TE/small RNAs and endosperm failure has only been noted in crosses between *A. thaliana* and *A. arenosa*. However, in these specific crosses, several potentially contributing factors are difficult to disentangle: these species differ in TE sequence divergence, TE abundance, and ploidy. To more directly associate differences in TE/small RNAs with the expression of postzygotic barriers, it would be preferable to exclude factors, such as ploidy, that might also contribute to isolation via effects unrelated to TE differences. One such approach would be to compare the expression of isolating barriers among groups of closely related (homoploid) species that differed in known TE load and/or sequence identity. Contrasting the mean strength, identity, and direction of reproductive isolation between TE divergent lineages with that between TE-similar lineages would provide a statistical assessment of the potential contribution of TE divergence (and misregulation) to the expression of species barriers and identify which isolation stages are differentially affected. These associations might also allow a finer resolution analysis as, depending on the species group, some species might only differ in TE copy number or sequence identity rather than differing in both. This would permit the effects of TE load and sequence identity to be evaluated independently. Finally, this approach would identify specific taxon pairs for which crossing evidence associating TE differences and isolating barriers is strongest. Such pairs could be targets for further detailed functional analysis.

A complementary strategy to understand the association between TE divergence and isolating barriers is to examine previously identified incompatibility QTL for the presence of TEs (or TE regulatory sequences). Fluorescent *in situ* hybridization (FISH) could be used to evaluate colocalization of TEs and incompatibility QTL, but this would rely on existing libraries of known TEs from the organism (or a closely related species) and *a priori* hypotheses of the importance of specific TEs. Positional cloning of QTL regions—itself a challenging empirical goal—could also facilitate examination of QTL for TE sequences.

### 4.3. Evaluating Repression of TEs in Hybrids

Finally, to unambiguously link reproductive isolating barriers with TE misregulation, it will be necessary to functionally connect specific developmental or reproductive problems in hybrids to TE derepression. To achieve this, a combined technique of methylation profiling and qPCR could be used to determine if TE transcripts (and specifically those known to differ between lineages) are active in the affected tissues. Methylation profiling can be achieved by using methyl-sensitive restriction enzymes on PCR products from DNA, but this requires a known TE sequence to develop appropriate primers. Bisulfite sequencing of DNA could provide an alternative approach, but a genomic DNA sequence is still needed to see which nucleotides have been converted after bisulfite treatment.

In this respect, profiling the endosperm appears to be the most straightforward initial step, as this tissue is relatively easily accessible via dissection from within the developing seed. For example, laser microdissection has already been used to specifically profile gene expression individually in the endosperm and embryo [78]; identical techniques could be used to isolate relevant tissues for siRNA and TE profiling. In comparison, to profile TE misregulation in the pollen, individual sperm cells must be dissected from their surrounding tissue, including the adjacent vegetative nucleus which is known to be demethylated (see above). Regardless, it is clear that established next generation and microdissection techniques can be used to address the specific question of whether divergent TEs are derepressed in developmentally abnormal hybrid tissues.

## 5. Conclusions

The idea that some reproductive isolating barriers (and therefore speciation processes) are the outcome of evolutionary conflicts has a long history but has been, until recently, mostly lacking in direct empirical support [4]. One such conflict is that between genomic parasites such as TEs and their hosts. Emerging mechanistic models of TE regulation via sRNA-mediated pathways provide renewed support for the hypothesis that TEs might be involved in the expression of interspecific reproductive barriers. Here, we have outlined several evolutionary corollaries that emerge from these mechanistic models, including the likely nonindependence of reproductive isolation acting at different stages, and the possible influence of particular demographic and life history factors on the relative susceptibility of lineages to hybrid problems based on TE misregulation. Some of these conditions themselves suggest further implications. For example, our inferences suggest that TE-mediated hybrid problems might be strongest at early stages of F1 development (e.g., during endosperm development) where lineage differences in TE load and identity can both contribute to hybrid problems. In comparison, F1 sterility is likely only influenced by differences in TE identity (see above) and requires additional conditions that seem restrictive, such as haploinsufficiency of siRNA-mediated TE suppression. (We know of no current evidence for or against this condition.) Other expectations about when and where TE misexpression might be most important are more challenging to predict at present. For example, differentiation of TEs is arguably more contingent on population subdivision than are some other potential hybrid incompatibilities; however, more theoretical and empirical attention will be necessary to address such questions conclusively.

Drawing from specific molecular models, in this review we have focused on mechanisms described in plants. Similar inferences could be tailored to RNA-mediated TE silencing mechanisms in animals. Indeed, both plants and animals appear to have similar small RNA silencing pathways; for example, while PIWI components have not been found in plants, divergent RNA polymerases that are responsible for small RNA amplification could replace PIWI proteins [63]. Still, as noted above, the lack of TE transposition in animal hybrids has created speculation as to whether TEs could play a role in the evolution of animal hybrid incompatibilities. Hybrid inviability associated with TE misregulation has not been observed in *Drosophila*, although the *Drosophila* studies that describe hybrid dysgenesis used lines from the same species that differ in the presence of relatively few TEs [6–8]. The involvement of TEs in *Drosophila* hybrid problems remains, therefore, to be established. We note, however, that the detection ability of TE-mediated hybrid problems might be greater in the endosperm because embryos can be “rescued” from failed endosperm (via independent culture), but *Drosophila* larvae cannot be rescued from failed eggs. In addition, the most detailed studies of hybrid problems in *Drosophila* (in the melanogaster-simulans group) are all crosses involving species with small genomes, and relatively few TEs. (Some *Drosophila* appear to have large genomes due to TEs, but these are not the species used to study speciation.) Still, it is possible that TEs are more relevant to plants; on the whole, plant genomes have many TEs, and even closely related species can differ substantially in their TE complement. As yet, it remains an open question whether TEs will be a “specialist” speciation mechanism or can apply broadly across sexually reproducing organisms.

Finally, we argue that, in order to more convincingly connect TE activity with postzygotic isolating barriers, three pieces of empirical evidence will be essential: lineage differences in TE load and/or identity; clear associations between this differentiation and the expression of hybrid incompatibilities; functional evidence for a mechanistic link between these two phenomena. These data can be obtained through current or rapidly emerging approaches in genetics and genomics. As such, the field is now well positioned to evaluate the connection, if any, between the misregulation of TEs and the expression of hybrid problems—a long-held, intriguing, but poorly assessed mechanism of speciation.
